# STIM1 dimers undergo unimolecular coupling to activate Orai1 channels

**DOI:** 10.1038/ncomms9395

**Published:** 2015-09-24

**Authors:** Yandong Zhou, Xizhuo Wang, Xianming Wang, Natalia A. Loktionova, Xiangyu Cai, Robert M. Nwokonko, Erin Vrana, Youjun Wang, Brad S. Rothberg, Donald L. Gill

**Affiliations:** 1 Department of Cellular and Molecular Physiology, The Pennsylvania State University College of Medicine, Hershey, Pennsylvania 17033, USA; 2 Beijing Key Department of Genetics and Developmental Biology, College of Life Sciences, Beijing Normal University, Beijing 100875, China; 3 Department of Biochemistry, Temple University School of Medicine, Philadelphia, Pennsylvania 19140, USA

## Abstract

The endoplasmic reticulum (ER) Ca^2+^ sensor, STIM1, becomes activated when ER-stored Ca^2+^ is depleted and translocates into ER–plasma membrane junctions where it tethers and activates Orai1 Ca^2+^ entry channels. The dimeric STIM1 protein contains a small STIM-Orai-activating region (SOAR)—the minimal sequence sufficient to activate Orai1 channels. Since SOAR itself is a dimer, we constructed SOAR concatemer–dimers and introduced mutations at F394, which is critical for Orai1 coupling and activation. The F394H mutation in both SOAR monomers completely blocks dimer function, but F394H introduced in only one of the dimeric SOAR monomers has no effect on Orai1 binding or activation. This reveals an unexpected unimolecular coupling between STIM1 and Orai1 and argues against recent evidence suggesting dimeric interaction between STIM1 and two adjacent Orai1 channel subunits. The model predicts that STIM1 dimers may be involved in crosslinking between Orai1 channels with implications for the kinetics and localization of Orai1 channel opening.

## Abstract (web summary)

STIM1, the endoplasmic reticulum calcium sensor, is activated in response to calcium store depletion and translocates to the ER–plasma membrane junctions to activate Orai1 channels. Here the authors present a new model of unimolecular coupling between STIM1 and Orai1 whereby STIM1 dimers may be involved in crosslinking Orai1 channels.

## Graphical abstract

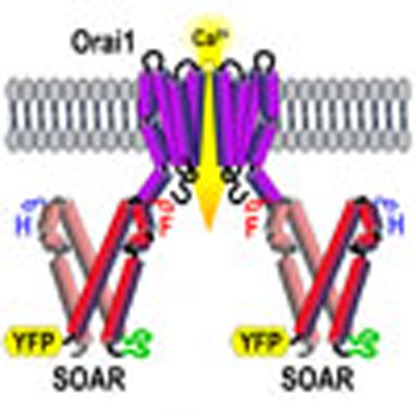

The widely expressed endoplasmic reticulum (ER) Ca^2+^ sensor protein, STIM1, undergoes an intricate activation process in response to Ca^2+^ store depletion, and translocates into ER–plasma membrane (PM) junctions where it tethers and activates PM Orai1 Ca^2+^ channels^1^. Ca^2+^ entering through Orai1 channels maintains Ca^2+^ homeostasis, sustains Ca^2+^ oscillations and mediates Ca^2+^ signals crucial for controlling gene expression^1^,^2^,^3^,^4^. Despite the significance of STIM1–Orai1 interactions in mediating cellular signals, considerable uncertainty and controversy surrounds the molecular mechanism and stoichiometry of the coupling process between STIM1 and Orai1 (refs 3, 4, 5).

From crystallographic data, the PM Orai1 channel appears to be a hexameric assembly of Orai1 subunits each of which is a four transmembrane-spanning protein arranged such that the six N-terminal membrane-spanning helices form a central pore within the hexamer^6^. STIM1 is a single transmembrane-spanning ER protein. Its luminal N terminus senses changes in stored Ca^2+^ through a pair of Ca^2+^-binding EF hand domains; the cytoplasmic C terminus includes a small, compact and highly conserved STIM-Orai-activating region (SOAR; 344–442)^7^ contained within the larger Ca^2+^ release activated Ca^2+^ (CRAC)-activating domain (CAD; 342–448)^8^ or Orai1-activating small fragment (233–450)^9^. Expressed alone, these domains are sufficient to fully activate the Orai1 channel. The crystal structure of the minimally functional unit, SOAR, reveals it to be a dimer, each peptide containing four α-helices^10^ (Fig. 1). The complete STIM1 protein also likely exists as a dimer in its resting state^9^,^11^, the SOAR sequences providing an important core locus for the dimeric interactions that link and organize the two STIM1 monomers^9^,^10^,^11^,^12^. Within the resting STIM1 dimer, the SOAR unit is occluded inside the large folded STIM1 cytoplasmic C terminus^12^. Sensing loss of ER Ca^2+^, the luminal STIM1 N termini rearrange within the dimer to trigger unfolding of the C-terminal domains in the STIM1 dimer, exposing the single dimeric SOAR region that can now bind to and activate the Orai1 channel^10^,^12^,^13^ (Fig. 1).

The nature of the coupling interaction between the activated STIM1 protein and the Orai1 channel is a crucial but unresolved question despite considerable scrutiny^3^,^4^,^5^. The stoichiometry of interaction between STIM1 and Orai1 is curiously variable^14^,^15^, with maximal Orai1 channel activation occurring when the STIM1:Orai1 ratio is 2:1 (refs 15, 16), that is, six dimers of STIM1 associated with one Orai1 hexameric channel. However, recent NMR studies, using isolated partial SOAR fragments and SOAR-binding C-terminal helices from Orai1, are interpreted to suggest that a bimolecular interaction in which a dimer of STIM1 binds across two adjacent Orai1 subunits in the hexameric Orai1 channel^17^,^18^,^19^, consistent with a 1:1 stoichiometry. We took a simple approach to understand the STIM1–Orai1 interaction, based on recent identification of a powerful point mutation (F394H) in the Orai1-binding site of STIM1, that completely prevents STIM1 binding to and hence activation of Orai channels^20^. Yet, this mutation does not cause any change in the resting state of STIM1 or its ability to undergo activation by store depletion, or to move into and be retained within ER–PM junctions^20^. Using a set of mutated concatemer–dimers of SOAR, our results unexpectedly reveal that only one of the two Orai1-activating sites in the dimeric SOAR molecule is required to fully activate the Orai1 channel. This explains how a 2:1 ratio of STIM1 to Orai1 can cause maximal channel activation. The other active site in the SOAR dimer is not required for Orai channel activation, but is available nonetheless. We suggest that this other available site may interact with and undergo inter-hexameric Orai1 channel crosslinking. Since the F394 residue is not within the coiled-coil region suggested to undergo dimeric interactions with Orai1 dimers^17^,^18^, our findings indicate a rather different view of the STIM1–Orai1 interface from that recently put forward.

## Results

### SOAR-F394H forms dimers but cannot bind or activate Orai1

The 100-amino-acid SOAR (or CAD) unit of STIM1 is sufficient for authentic activation of the Orai1 channel^7^,^8^. SOAR combines tightly with the Orai1 C terminus of Orai1, although the N terminus of Orai1 has also been implicated in the interaction^8^,^21^,^22^. From recent work, we noted that while the SOAR sequences of STIM1 and STIM2 are almost identical, a crucial phenylalanine residue (394), prominently located at the apex of the SOAR–Orai1-coupling site of STIM1, is replaced in STIM2 with a leucine accounting for a considerable reduction in the efficacy of interaction of STIM2 with Orai1 compared with STIM1 (ref. 20). Substituting this phenylalanine with histidine (F394H) in the full-length STIM1 molecule results in a complete loss of Orai1 activation by STIM1 (ref. 20). The STIM1-F394H mutant is distributed throughout the ER in resting cells and is activated and moves into ER–PM junctions on Ca^2+^ store depletion, just as for wild-type STIM1. However, it neither binds to nor activates the Orai1 channel^20^. This single point mutation within the Orai1-interacting site is used here as an important tool to dissect the coupling interaction between STIM1 and Orai1.

**Figure 2:**
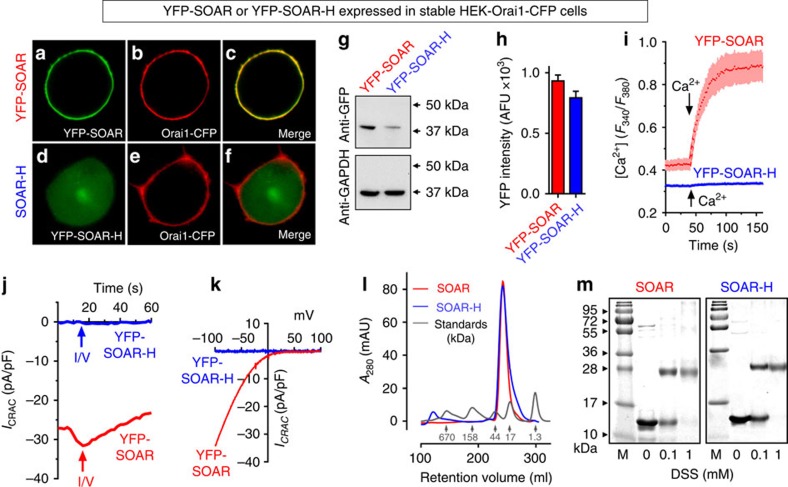
The F394H mutation in SOAR prevents its binding to and gating of Orai1 channels without altering its size or ability to dimerize. SOAR constructs were transiently expressed in HEK-Orai1-CFP stable cells. The YFP-labelled wild-type SOAR protein (YFP-SOAR) is exclusively PM localized where its distribution is superimposable with Orai1 (**a**–**c**). In contrast, the YFP-tagged F394H mutant (YFP-SOAR-H) is exclusively cytosolic (**d**–**f**). (**g**) Expression of YFP-SOAR and YFP-SOAR-H detected with GFP antibody. (**h**) Average YFP fluorescence intensity (arbitrary fluorescence units, AFUs) of cells shown in **i**±s.e.m. (**i**) Fura-2 ratiometric Ca^2+^ responses in HEK-Orai1-CFP cells transiently expressing similar levels of YFP-SOAR (*n*=32) or YFP-SOAR-H (*n*=8). Results are means±s.e.m. and representative of three independent repeats. Constitutive Ca^2+^ entry was measured in nominally Ca^2+^-free medium and after addition of 1 mM Ca^2+^ (arrow). (**j**) Whole-cell patch-clamp recording of *I*_CRAC_ in HEK-Orai1-CFP cells transfected with YFP-SOAR or YFP-SOAR-H. Bath solutions contained 20 mM Ca^2+^ and cytosolic-free Ca^2+^ was maintained at 90 nM. (**k**) *I*–*V* relationship from cells shown in j. (**l**) Size-exclusion chromatogram of purified wild-type SOAR (red) and F394H SOAR mutant (blue), showing both elute from a Superdex-200 16/60 column as a single peak at ∼245 ml, corresponding to 25 kDa (monomeric SOAR, is 12 kDa). Protein standards (grey) correspond to molecular weights of (i) 670, (ii) 158, (iii) 44, (iv) 17 and (v) 1.3 kDa. (**m**) Chemical crosslinking of purified SOAR subunits with DSS. Coomassie blue-stained SDS–PAGE of SOAR wild-type (left) and SOAR-F394H (right) after incubation with 0, 0.1 and 1 mM DSS at room temperature for 30 min. M: protein marker.

Our studies utilized a series of SOAR-based constructs (Supplementary Fig. 1). As shown in Fig. 2, introduction of the F394H point mutation into the SOAR fragment of STIM1 entirely precludes its Orai1-coupling function. Transiently expressed, wild-type yellow fluorescent protein (YFP)-SOAR was bound avidly to Orai1-cyan fluorescent protein (CFP) channels stably expressed in HEK cells (HEK-Orai1-CFP cells) with complete overlap of fluorescence (Fig. 2a–c). In contrast, YFP-SOAR-H (the single F394H mutation) expressed transiently in the same cell type was devoid of any binding and remained cytosolic with no CFP-Orai1 overlap (Fig. 2d–f). Although western analysis revealed lower YFP-SOAR-H expression than YFP-SOAR (Fig. 2g), this reflected fewer YFP-SOAR-H-transfected cells rather than lower expression per cell. In populations of cells expressing equal levels of YFP-SOAR or YFP-SOAR-H (Fig. 2h), we compared Ca^2+^ entry following Ca^2+^ add-back to fura-2-loaded HEK-Orai1-CFP cells (Fig. 2i). Whereas YFP-SOAR-expressing cells had substantial constitutive Ca^2+^ entry without depleting stores, cells with equally expressed YFP-SOAR-H were devoid of Ca^2+^ entry. Patch-clamp analysis of CRAC current (*I*_CRAC_) without store depletion revealed substantial constitutive current in YFP-SOAR-expressing HEK-Orai1-CFP cells (Fig. 2j) with *I*/*V* profile typical of the inwardly rectifying properties of the opened Orai1 channel (Fig. 2k). In contrast, cells equally expressing YFP-SOAR-H fluorescence had no measureable CRAC current under the same conditions (Fig. 2j,k).

Evidence suggests that both the intact resting STIM1 protein as well as SOAR and other C-terminal fragments derived from STIM1, exist as dimers^7^,^10^,^11^,^12^,^23^,^24^. We considered whether the F394H mutation might alter the ability of SOAR to dimerize. Size-exclusion chromatography on a Superdex-200 column, revealed that both SOAR and SOAR-H elute with a single identical peak corresponding to a molecular weight of ∼25 kDa (the SOAR monomer is 12 kDa) (Fig. 2l). We undertook chemical crosslinking of purified SOAR and SOAR-H using 0, 0.1 and 1 mM disuccinimidyl suberate (DSS) for 30 min at room temperature (Fig. 2m). Coomassie blue-stained SDS–polyacrylamide gel electrophoresis revealed that the SOAR (left) and SOAR-H bands (right) both partially shifted to a dimeric crosslinked product at 0.1 mM DSS, and entirely to a dimeric form using 1 mM DSS, with no evidence of larger multimers. Thus, SOAR exists as a dimer and the F394H mutation does not alter this dimer formation nor result in any other association or alteration in the size of the SOAR dimer.

### SOAR dimers containing one F394H mutation still bind Orai1

**Figure 3:**
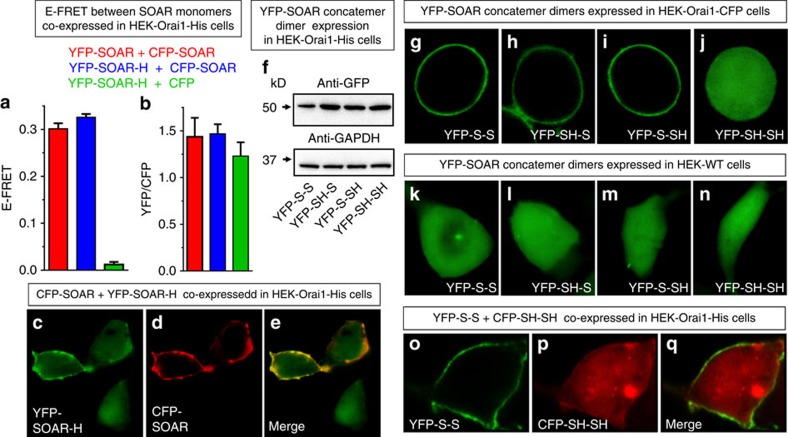
Expression, distribution and interactions of SOAR monomers and SOAR concatemer–dimers. (**a**) Summary of E-FRET values between YFP-SOAR and CFP-SOAR (red, *n*=21), or YFP-SOAR-H and CFP-SOAR (blue, *n*=45), or YFP-SOAR-H and CFP (green, *n*=14), transiently co-expressed in HEK-Orai1-His stable cells. Analysis was restricted to the near-PM area. (**b**) YFP/CFP ratios of the near-PM-expressed proteins in the cells used for E-FRET in **a**. Results are means±s.e.m. of three independent repeats. (**c**–**e**) YFP-SOAR-H and CFP-SOAR co-transfected in HEK-Orai1-His cells. The view shows three cells all expressing YFP-SOAR-H (**c**), but only two cells co-expressing CFP-SOAR (**d**). The merged image (**e**) reveals some YFP-SOAR-H localized to the PM in the two cells co-expressing CFP-SOAR; YFP-SOAR-H remains cytosolic in the cell not co-expressing CFP-SOAR. (**f**) Western analysis of the four SOAR concatemer–dimers shown, transiently expressed in stable HEK-Orai1-His using an anti-GFP antibody. (**g**–**j**) Distribution of the same four SOAR YFP-tagged concatemer–dimers transiently expressed in stable HEK-Orai1-CFP cells: (**g**) wild-type dimer YFP-S-S, (**h**,**i**) heterodimer mutants YFP-SH-S and YFP-S-SH, and (**j**) the homo-mutant dimer YFP-SH-SH; dimers are defined in Supplementary Fig. 1. Distribution of Orai1-CFP in the same cells is shown in Supplementary Fig. 2a–h. (**k**–**n**) Distribution of the same four concatemer–dimers expressed this time in HEK-WT cells. (**o**–**q**) Co-transfection of the wild-type YFP-S-S concatemer–dimer with the double-mutant CFP-SH-SH concatemer–dimer in HEK-Orai1-His cells.

A further question was whether the SOAR molecule still undergoes self-association when expressed in cells and, more importantly, how mutations in each of the two active sites of the SOAR dimer might alter coupling with and activation of the Orai1 channel. Initially, we examined Förster resonance energy transfer (FRET) between YFP- and CFP-labelled derivatives of SOAR, undertaking three-cube FRET imaging to determine instrument-independent FRET efficiency (E-FRET)^25^. In HEK-Orai1-His cells, E-FRET between transiently co-expressed YFP-SOAR and CFP-SOAR was almost identical to that between YFP-SOAR-H and CFP-SOAR (Fig. 3a) in cells in which the YFP/CFP ratio was within a narrow range (Fig. 3b). These E-FRET levels were far in excess of those between YFP-SOAR-H and unconjugated CFP expressed in cells (Fig. 3a). Having shown that the CFP-SOAR and YFP-SOAR-H could undergo interaction within the cell, we considered whether the mixed SOAR dimer would still interact with Orai channels in the PM. We predicted that this interaction would not occur or at least would be hindered based on the recent model that STIM1 dimers undergo a bimolecular interaction with Orai channels^17^,^18^. We co-expressed both CFP-SOAR and YFP-SOAR-H in HEK-Orai-His cells. The images of three adjacent cells (typical of cells on this coverslip and in other independent experiments) are shown in Fig. 3c–e. All three cells expressed YFP-SOAR-H (Fig. 3c). Two of these cells co-expressed CFP-SOAR, which was clearly localized to the PM (Fig. 3d). In the two cells that expressed both constructs, both were localized to the PM even though some YFP-SOAR-H remained in the cytoplasm. However, in the single cell that expressed only YFP-SOAR-H, all the fluorescence remained cytoplasmic (Fig. 3e). These results indicate that YFP-SOAR-H can associate with CFP-SOAR and that the complex itself is able to bind to Orai1.

**Figure 4:**
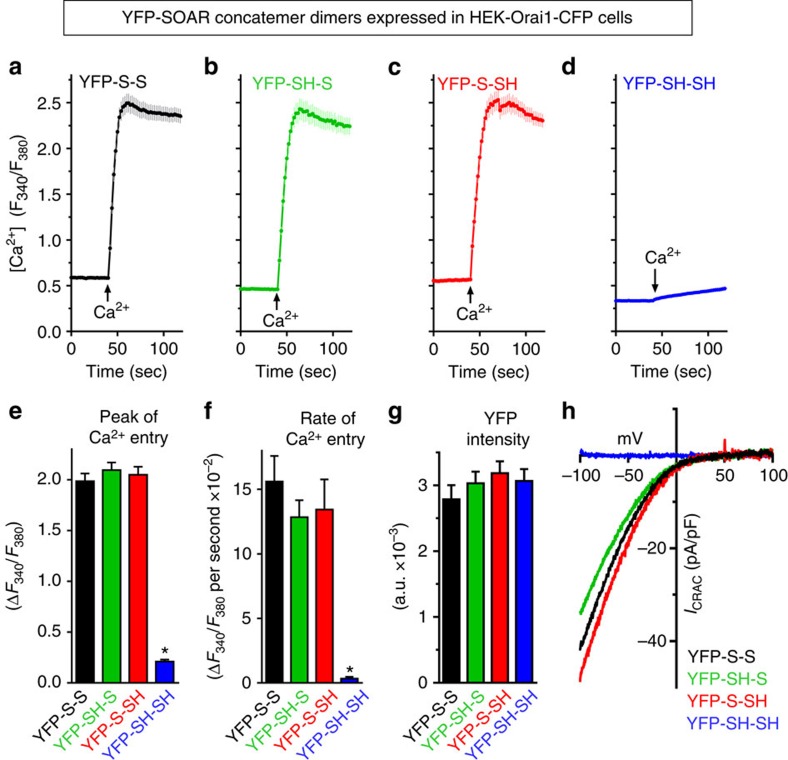
The three SOAR concatemer–dimers, YFP-S-S, YFP-SH-S and YFP-S-SH are functionally identical. Fura-2 ratiometric Ca^2+^ responses in stable HEK-Orai1-CFP cells transiently expressing the same levels of either: (**a**) YFP-S-S (43 cells); (**b**) YFP-SH-S (50 cells); (**c**) YFP-S-SH (49 cells); (**d**) YFP-SH-SH (48 cells). Constitutive Ca^2+^ entry was measured using cells in nominally Ca^2+^-free solution, replaced with 1 mM Ca^2+^ solution (Ca^2+^). (**a**–**d**) Results are means of the cell numbers indicated±s.e.m. and are representative of three independent repeats. (**e**) Statistics for average peak of constitutive Ca^2+^ entry shown in **a**–**d** ,*P<0.001 from YFP-S-S; (**f**) statistics for Ca^2+^ entry rates shown in **a**–**d**, *P<0.001 from YFP-S-S; (**g**) average YFP fluorescence intensity (arbitrary fluorescence units; AFUs) of cells used in **a**–**d**). (**e**–**g**) Results are means±s.e.m. of three independent experiments. (**h**) *I*/*V* relationship of whole-cell *I*_CRAC_ measurements for YFP-S-S (black), YFP-SH-S (green), YFP-S-SH (red) or YFP-SH-SH (blue) transiently expressed in HEK-Orai1-CFP stable cells. Quantification of current densities for the three active concatemer–dimers are given in Supplementary Fig. 3.

**Figure 5:**
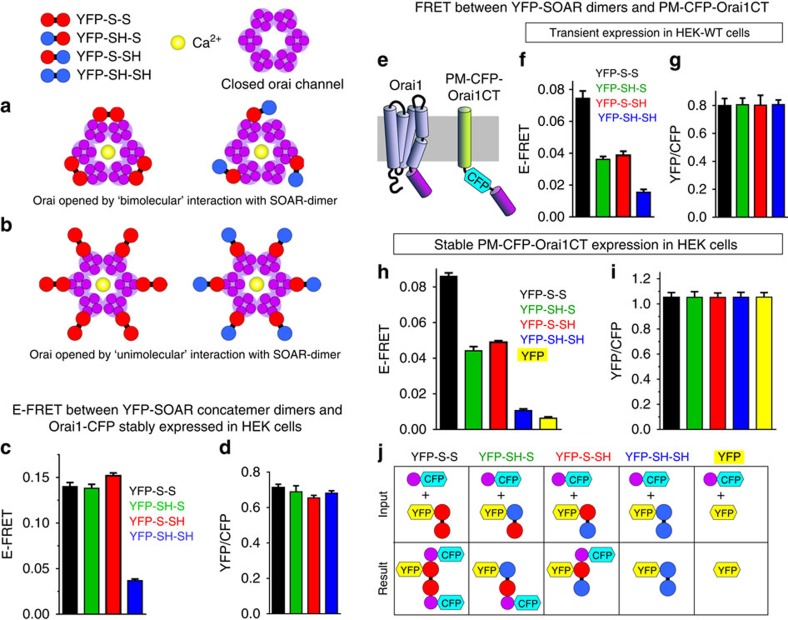
Evidence for a unimolecular interaction between the SOAR dimer and Orai1. Two possible models for SOAR–Orai1 interactions: (**a**) bimolecular binding of a homomeric SOAR dimer (red) to two adjacent Orai1 subunits is required to open the hexameric Orai1 channel (purple). (**b**) Unimolecular binding of a single monomer within the SOAR dimer (red) to a single Orai1 subunit is sufficient to open the hexameric Orai1 channel (purple). Changes in the association of heterodimers of SOAR comprising one WT and one F394H monomer (red/blue) occurring in the bimolecular (**a**, right) and unimolecular (**b**, right) models. (**c**) Near-PM values of E-FRET between Orai1-CFP in HEK-Orai1-CFP stable cells, and transiently expressed YFP-S-S (black; *n*=141), YFP-SH-S (green, *n*=243), YFP-S-SH (red, *n*=213) or YFP-SH-SH (blue, *n*=206). (**d**) YFP/CFP ratios of the near-PM-expressed Orai1-CFP and YFP dimers in the cells used for E-FRET in **c**. (**e**) Cartoon of Orai1 protein (left) and PM-CFP-Orai1CT construct (right) comprising C terminus of Orai1 (267–301) attached to CFP and a single PM transmembrane-spanning helix. (**f**) Near-PM E-FRET between PM-CFP-Orai1CT transiently expressed in HEK-WT cells and transiently expressed SOAR concatemer–dimers: YFP-S-S (black; *n*=51); YFP-SH-S (green; *n*=55); YFP-S-SH (red; *n*=39); and YFP-SH-SH (blue; *n*=85). (**g**) YFP/CFP ratios of near-PM-expressed Orai1-CFP and YFP dimers in the cells used (**f**). (**h**) Near-PM E-FRET between PM-CFP-Orai1CT stably expressed HEK cells and transiently expressed SOAR concatemer–dimers: YFP-S-S (black; *n*=141); YFP-SH-S (green; *n*=137); YFP-S-SH (red; *n*=389); YFP-SH-SH (blue; *n*=202); and YFP alone (yellow; *n*=202). (**i**) YFP/CFP ratios of near-PM-expressed Orai1-CFP and YFP dimers in the cells used in **h**. In all cases (c–i), results are means±s.e.m. of three independent experiments. (**j**) Interpretation of interactions between CFP-tagged Orai1CT (purple) and each of the four YFP-tagged SOAR dimer–concatemers (or YFP alone) used in the E-FRET experiments shown in **h**. Top row shows input reactants for each condition; bottom row shows resulting interactions. Each WT SOAR monomer (red) of the SOAR dimers is shown to independently bind a single Orai1CT molecule. F394H SOAR monomers (blue) do not bind Orai1CT.

This unexpected result prompted us to more directly examine whether the SOAR dimer underwent a unimolecular or bimolecular interaction with the Orai1 channel. For this purpose, we made a series of four YFP-tagged SOAR concatemer–dimer constructs in which two SOAR monomers were joined through a 24-amino-acid linker. As shown in Supplementary Fig. 1, these dimeric constructs comprised either two wild-type SOAR units (YFP-S-S), a combination of wild-type and F394H-mutated SOAR units (YFP-S-SH and YFP-SH-S), or two F394H-mutated SOAR units (YFP-SH-SH). Western analysis revealed that expression of each of the four dimers in HEK-Orai1-His cells was highly consistent (Fig. 3f), as were YFP fluorescence levels in individual cells within each population (Figs 4 and 5). The wild-type homodimer (YFP-S-S) was exclusively associated with the PM, as expected (Fig. 3g). So, also were the two heterodimers, YFP-SH-S (Fig. 3h) and YFP-S-SH (Fig. 3i), despite having only one active Orai1-binding site. In contrast, the homo-mutant dimer (YFP-SH-SH), devoid of Orai1-binding sites, was exclusively cytosolic (Fig. 3j). The association of YFP-S-S, YFP-SH-S and YPF-S-SH with the PM was clearly driven by interaction with Orai1 since there was almost perfect overlap with Orai-CFP fluorescence (Supplementary Fig. 2a–h). More significantly, there was no PM association of these constructs in wild-type HEK cells (that is, not overexpressing Orai1-CFP) (Fig. 3k–m). The cytosolic distribution of YFP-SH-SH was unaffected by the presence or absence of Orai1 (Fig. 3j,n; Supplementary Fig. 2d,h). We also examined co-expression of the Orai1-interacting YFP-S-S construct together with the Orai1 non-interacting CFP-tagged version of SH-SH (CFP-SH-SH; Fig. 3o–q). There was clearly no overlap of fluorescence (Fig. 3q), indicating there were no dimer–dimer interactions that might allow the wild-type SOAR dimer to bring the homo-mutant dimer to the membrane, in contrast to the results using co-expressed monomers (Fig. 3c–e). Indeed, using HEK-WT cells, we directly compared the E-FRET between co-expressed YFP-SOAR and CFP-SOAR monomers with E-FRET between co-expressed YFP-S-S and CFP-S-S concatemer–homodimers, as well as between YFP- and CFP-tagged heterodimers (Supplementary Fig. 2i,j). Thus, there was no evidence for dimer–dimer interactions and no support for the earlier observation that CAD can form tetramers^8^.

### Heterodimeric SOAR with one F394H mutation activates Orai1

These results provide evidence that a unimolecular interaction of the SOAR dimer is sufficient for association with the Orai1 channel. An important further question was whether this unimolecular coupling of SOAR was sufficient for Orai1 channel activation. We compared Ca^2+^ entry within HEK-Orai1-CFP cells that were transfected with each of the four concatemer–dimers, YFP-S-S, YFP-SH-S, YFP-S-SH or YFP-SH-SH (Fig. 4a–g; Supplementary Fig. 2k). Under identical Ca^2+^ add-back conditions, and using cells expressing the same levels of each concatemer–dimer, the results reveal a remarkable similarity in the actions of YFP-S-S, YFP-SH-S and YFP-S-SH on Ca^2+^ entry (Fig. 4a–c). In contrast, the YFP-SH-SH construct was unable to induce any constitutive Ca^2+^ entry (Fig. 4d). The levels of peak Ca^2+^ entry mediated by YFP-S-S, YFP-SH-S and YFP-S-SH were almost identical (Fig. 4e). Moreover, we could resolve the initial rate of Ca^2+^ entry following Ca^2+^ add-back, and this too remained very similar for the same three constructs (Fig. 4f). The expression levels of YFP fluorescence in each of the transfected cell populations for the four constructs (Fig. 4g) were all very similar. Examining the CRAC current activity in HEK-Orai1-CFP cells expressing the YFP-S-S, YFP-SH-S and YFP-S-SH concatemer–dimers (Fig. 4h), we observed high and statistically similar levels of constitutive current for all three constructs (Supplementary Fig. 3), but no current in cells expressing the YFP-SH-SH homo-mutant construct (Fig. 4h). The almost identical *I*/*V* profiles of channel activity induced by the YFP-S-S, YFP-SH-S and YFP-S-SH constructs indicated there was no alteration in the reversal potential or rectification properties of the Orai1 channel.

A number of studies have shown that SOAR must be presented to Orai1 as a dimer. In one study, Li, *et al.*^16^ determined that a monomer SOAR unit concatenated with Orai1 resulted in little channel opening compared with Orai1 concatenated with a SOAR dimer (Orai1-S-S). We wondered whether inducing the close proximity of the SOAR dimer to Orai1 in such a concatemer might alter the requirement for only a single functional monomer within the dimer. Using similar Orai1-linked SOAR–SOAR concatemers, we observed that the F394H mutation on either of the two SOAR units resulted in constitutive Ca^2+^ entry almost identical to the construct containing two wild-type SOAR units (Supplementary Fig. 4). Thus, constraining the proximity of the SOAR dimer towards Orai1 does not alter the requirement for only a single functional SOAR unit to activate the channel.

### The SOAR dimer interacts with two Orai1 channel C termini

The unexpected picture to emerge from the cellular distribution (Fig. 3) and Orai1 functional coupling data (Fig. 4) is that a dimeric SOAR molecule with only one Orai1-binding site induces identical functional coupling to activate the Orai1 channel as a dimeric SOAR molecule with two Orai1-binding sites. We considered two basic models for the interaction of SOAR dimers with the multimeric (likely hexameric) Orai1 channel. First, a ‘bimolecular' interaction in which SOAR dimers interact with two adjacent Orai1 subunits (Fig. 5a, left), and second, a ‘unimolecular' interaction in which only one of the two Orai1-binding sites of the SOAR dimer interacts with each Orai1-binding site (Fig. 5b, left). Although the above data support the second model, they do not exclude the first. Thus, in the bimolecular model, even though both SOAR dimer units might normally bind to a dimer of Orai1, it is possible that only one SOAR interaction with each Orai1 dimer is sufficient for binding and activation of the channel. In this case, we might expect the heterodimers of SOAR to undergo some altered configurational arrangement in the SOAR–Orai1 interaction (Fig. 5a, right). We examined E-FRET between each of the YFP-labelled SOAR concatemer–dimers and Orai1-CFP in the stable HEK-Orai1-CFP cell line. As seen in Fig. 5c, E-FRET levels between Orai1-CFP and YFP-SH-S or YFP-S-SH were almost the same as E-FRET between Orai-CFP and YFP-S-S. E-FRET between Orai1-CFP and the YFP-SH-SH construct was much lower, as expected. To ensure comparability between the E-FRET measurements, analyses were restricted to cells within a narrow range of YFP/CFP ratios (Fig. 5d). Thus, there is no detectable distinction between the interactions of the ‘divalent' as opposed to the ‘univalent' forms of the SOAR dimers, with Orai1, and no evidence to support any alteration in the SOAR–Orai1 interaction, providing further support for a unimolecular interaction that is uninfluenced by mutation of one of the two SOAR units in the SOAR dimer (Fig. 5b, right).

We sought to provide an independent means of verifying the unexpected unimolecular interaction model. We examined whether both sites on the SOAR molecule were available to independently interact with Orai1-binding sites for STIM1. Studies reveal that although STIM1 or SOAR are thought to undergo interactions with both the N- and C-terminal sequences of Orai1, the strongest interactions are with the helical cytosolic Orai1 C terminus (amino acids 267–301)^8^,^21^,^26^,^27^. Thus, we assessed interactions of the YFP-SOAR concatemer–dimers with a new construct (Fig. 5e) comprising the Orai1 C terminus (267–301) linked via CFP to a PM-directed single transmembrane-spanning helix (PM-CFP-Orai1CT)^28^. We confirmed the PM localization as well as the orientation of the PM-CFP-Orai1CT using a fluorescence protease protection assay^29^ as shown in Supplementary Fig. 5. Initially, we transiently co-expressed this construct with each of the four SOAR concatemer–dimers in HEK cells (Fig. 5f,g). Later, we used a stable cell line expressing the PM-CFP-Orai1CT construct in which each of the four concatemer–dimers were transiently expressed (Fig. 5h,i). In both cases, the results reveal that the E-FRET between YFP-S-S and PM-CFP-Orai1CT was approximately twice the E-FRET levels seen using either YFP-SH-S or YFP-S-SH (Fig. 5f,h). The E-FRET levels with YFP-SH-SH were much lower and very close to background FRET observed with cells expressing YFP alone (Fig. 5h). In both experiments, E-FRET analyses were performed on large cell populations with a very narrow range of YFP/CFP ratios (Fig. 5g,i). The E-FRET results, explained in Fig. 5j, indicate that the YFP-S-S construct is able to bind two PM-CFP-Orai1CT molecules, hence the CFP donor can transfer twice the energy to the YFP acceptor. The YFP-SH-SH construct does not interact with the PM-CFP-Orai1CT and this E-FRET value represents a background level that is only slightly higher than the level observed with unconjugated cytosolic YFP. The two heterodimers, YFP-SH-S and YFP-S-SH, each bind only one PM-CFP-Orai1CT, and their E-FRET levels correspond to half the energy transfer of the YFP-S-S, thus their E-FRET levels lie approximately mid-way between YFP-S-S and YFP-SH-SH. These data indicate that the two SOAR units within the SOAR dimer are each able to independently interact with one Orai1-binding site and further argue against the bimolecular coupling of SOAR within a single Orai1 channel. The results also allow us to speculate and make some predictions on the nature of the STIM1–Orai1 coupling interface as discussed below.

## Discussion

The results in this study provide strong evidence that the dimeric STIM1 molecule need to undergo only a monomeric interaction with Orai1 to bind to and activate its channel activity. SOAR appears to always exist as a dimer whether expressed alone or within full-length STIM1. Mutations in those residues that mediate dimerization between the two monomers, profoundly alter the ability of SOAR to bind to and activate Orai1 channels^10^,^17^,^19^. However, such mutations do not necessarily cause dissociation of the dimer—instead they may interfere with the secondary structure of the SOAR dimer without dimer dissociation. Indeed, a recent report described the R429C loss of function STIM1 mutation in human patients^19^. This residue in the Sα4 helix of SOAR was revealed from crystallization data to mediate SOAR interdimer hydrogen bonding^10^. The mutation in whole STIM1 was shown to not only curtail Orai1 coupling, but also to unfold the entire STIM1 C terminus allowing its K-rich region to be exposed and for the mutated STIM1 molecule to constitutively move into ER–PM junctions^19^. The R429C mutation clearly altered the secondary structure of SOAR as determined by circular dichroism spectral analysis and thermal stability. However, it did not in fact dissociate the dimer. This provides good evidence that the structural integrity of SOAR is crucial not only for Orai1 coupling but also for maintaining the structure of the entire STIM1 molecule. The STIM1 R429C mutation likely has a greater effect on the structural integrity of the STIM1 molecule as compared with the F394H mutation. Thus, in contrast to the R429C mutation, STIM1 containing the F394H mutation is in a non-activated state, and is still able to respond to store depletion, become aggregated and move into ER–PM junctions^20^. It is deficient only in functional coupling with Orai1 because it lacks a crucial component of the physical coupling interaction. An earlier report using concatenated Orai1-S-S constructs^16^ revealed that the LQ347/348AA mutation introduced in only one of the two SOAR units resulted in substantial loss of Orai1 activation. In contrast, we reveal that the F394H mutation in only one of the two SOAR units in the same Orai1-S-S construct, did not affect Orai1 channel activation (Supplementary Fig. 4). It was shown in the SOAR crystallography studies that L347, like R429, is another residue critical for SOAR interdimer interactions^10^. Thus, its mutation may lead to significant structural perturbation of the SOAR dimer in a manner analogous to mutation of R429.

The critical question of whether both of the two identical active sites in the SOAR dimer moiety presented to Orai1 by STIM1 are required has not been previously addressed. Our finding that only one site is required has important implications in understanding the stoichiometry and geometry of STIM–Orai1 interactions. The recent bimolecular Orai1-binding model, by which SOAR dimers interact with the C termini of two adjacent Orai1 subunits, is based on NMR studies utilizing an isolated peptide from STIM1 (residues 312–387, the Sα1 helix of SOAR1 extended N terminally by 22 residues) and a 21 amino acid helical C-terminal sequence from Orai1 (refs 17, 18). But, unlike SOAR, the 312–387 STIM1 fragment does not have any ability to bind to or activate Orai1 channels. Nor, importantly, does it include the F394 residue we reveal to be critical to STIM1–Orai1 interaction and channel opening. Moreover, one of the two segments of the 312–387 STIM1 peptide purported to interact with the Orai1 C-terminal peptides^17^ is the exact segment shown in the crystallographic SOAR studies to be the SOAR–SOAR interface and critical to SOAR dimerization^10^. Hence, the dimer–dimer interaction model requires unfolding of the SOAR Sα1 helix from the Sα4 helix and the unravelling of SOAR. This would be incompatible with the data indicating a strong requirement for the structural integrity of the SOAR dimer.

The emerging unimolecular model (Fig. 5b) raises the question of the utility of the second Orai1-binding site on the SOAR dimer. Our finding that a single SOAR dimer is able to simultaneously bind to two C termini of Orai1 channels raises the intriguing possibility that the SOAR dimer might bridge between Orai1 hexamers. Significantly, in studies using the CAD sequence from STIM1 (almost identical to SOAR), Park *et al.*^8^ provided strong evidence from *in vitro* electron microscopic measurements, that CAD mediates crosslinking of Orai1 channels into clusters. Although clustering was not a requirement for channel activation by CAD, electron microscopy revealed CAD induced association of up to 20 or more channels within a single cluster. Given this information, and considering the unimolecular coupling model suggested here as well as the evidence that a dimer of SOAR is able to independently interact with two Orai1 C-terminal-binding sites, we speculate that STIM1 dimers may have the ability to induce crosslinking and clustering of Orai1 channels as shown in Supplementary Fig. 6.

Last, the evidence that the STIM1–Orai1 interaction has a variable stoichiometry with maximal activation involving two STIM1 molecules per Orai1 molecule^14^,^15^,^16^ is compatible with the unimolecular STIM1 coupling model presented here in which six dimers of STIM1 can interact with a single hexameric Orai1 channel (Fig. 5b). In contrast, the bimolecular STIM1 interaction model^17^,^18^ does not fit with such a 2:1 STIM1:Orai1 stoichiometry. If clustering between Orai1 channels resulted from SOAR-induced crosslinking as reported earlier^8^, a hypothetical lattice of Orai1 channels would have a stoichiometry of between 1 and 2 STIM1 per Orai1 channel depending on cluster size (Supplementary Fig. 6), compatible with the variable stoichiometry data revealing that Orai1 channel activation occurs with a ratio of between 1 and 2 SOAR proteins per Orai1 protein^16^. Under conditions where activated STIM1 was limited, less than a full complement of six STIM1 dimers bound to an Orai1 hexameric channel could result in partial channel activation. The concept of a STIM1-induced Orai1 lattice is provocative and as yet unsubstantiated, but could have important implications in channel activation and deactivation kinetics, and in assuring localized Ca^2+^ entry signals, crucial for the downstream effects of store-operated Ca^2+^ entry^30^,^31^. Investigation of possible clustering of Orai1 channels would provide an interesting focus for further studies. However, the significance of the current work is to provide strong evidence for a unimolecular coupling model as an important paradigm in STIM1–Orai1 activation. Such information militates against the recently adopted model of a dimeric interaction between STIM1 dimers and pairs of adjacent Orai1 subunits, and provides an important basis for understanding the molecular mechanism of STIM1-induced Orai1 channel gating.

## Methods

### DNA constructs

All monomer SOAR (WT or F394H mutants) and concatemeric–dimer SOAR constructs (Supplementary Fig. 1) were made by Mutagenex, NJ. Monomer SOAR was inserted within pEYFP-C1 or pECFP-C1 (Clontech) between XhoI/EcoRI as previously described^32^. Concatemeric–dimer SOAR (YFP-S-S) and mutated derivatives (YFP-S-SH, YFP-SH-S and YFP-SH-SH) were subsequently inserted between XhoI/KpnI sites. The F394H mutation was introduced using the QuikChange Lightning Site-Directed Mutagenesis Kit (Agilent Cat No. 210518). A 72-bp linker 5′-GGCGGCTCTGGAGGTAGCGGAGGTGGAATTCTGCAGTCGAGGGGTGGATCCGGTGGGTCCGGCGGATCCGGC-3′ (translated as the 24 amino acids GGSGGSGGGILQSRGGSGGSGGSG) was used in concatemer–dimer SOAR constructs. All constructs (YFP-SOAR, YFP-SOAR-H, YFP-S-S, YFP-SH-S, YFP-S-SH, YFP-SH-SH and CFP-SH-SH) were confirmed by sequencing before transfection. The construct PM-CFP-Orai1CT (including the Orai1 C-terminal sequence, 267–301) was made using the overlap extension PCR technique^33^ with primer pairs 5′-GGCATGGACGAGCTGTACAAGAAGCTTGCTGACCGACAGTTCCAGGAGCTCA-3′ and 5′-ACAGCTCATCCTTAAGTCGACTCGAGATGCGCTAGGCATAGTGGCTGCCGGG-3′ and PM-CFP-FRB template^28^ (a gift from M. K. Korzeniowski, Cornell University and T. Balla, NICHD). The intramolecular pEYFP-ECFP construct used to determine the G-parameter for E-FRET calculations^25^ was made by inserting ECFP into the pEYFP-C1 vector (Clontech) at HindIII/BamHI. The Orai1-S-S construct was a gift from Dr Tao Xu (Institute of Biophysics, Chinese Academy of Sciences, Beijing).

### Cell culture and transfection

HEK-WT cells were maintained in DMEM (Mediatech) supplemented with 10% fetal bovine serum, penicillin (100 U ml^−1^) and streptomycin (100 μg ml^-1^; Gemini Bioproducts, CA), and cultured in 5% CO_2_ at 37 °C (ref. 34). Stable cell lines including HEK-Orai1-CFP, HEK-Orai1-His and HEK-PM-CFP-Orai1CT were maintained in DMEM with fetal bovine serum as above together with G418 (final concentration, 100 μg ml^-1^). All transfections were achieved by electroporation at 180 V, 25 ms in 4-mm cuvettes (Molecular BioProducts) using the Bio-Rad Gene PulserXcell system in OPTI-MEM medium. All experiments were performed 18–24 h after transfection.

### Cytosolic Ca^2+^ measurements

Cytosolic Ca^2+^ levels were measured by ratiometric imaging^35^ using fura-2-loaded cells 18–24 h after transfection with monomer or concatemer–dimer SOAR constructs as described. To protect cells from any constitutive Ca^2+^ entry mediated by SOAR monomer or SOAR dimer–concatemer transfection, loading of fura-2 and imaging were performed in Ca^2+^-free solution (mM): 107 NaCl, 7.2 KCl, 1.2 MgCl_2_, 11.5 glucose, 20 Hepes-NaOH, pH 7.2. Addition of 1 mM CaCl_2_ was made as indicated in figures. For dye loading, cells were treated with 2 mM fura-2/AM for 30 min at room temperature, and then transferred into fura-2-free solution for a further 30 min. Fluorescence ratio imaging was measured by a Leica DMI 6000B fluorescence microscope and Hamamatsu ORCA-Flash 4 camera controlled using Slidebook 6.0 software (Intelligent Imaging Innovations; Denver, CO). Consecutive excitation at 340 nm (F340) and 380 nm (F380) was applied every 2 s and emission fluorescence was collected at 505 nm. Intracellular Ca^2+^ levels are shown as *F*_340_/*F*_380_ ratios obtained from groups of >35 single cells on coverslips. All Ca^2+^ imaging experiments were performed at room temperature and representative traces of at least three independent repeats are shown as mean±s.e.m.

### Förster resonance energy transfer measurements

To determine FRET signals between stably expressed Orai1-CFP or PM-CFP-Orai1CT and transiently expressed YFP-tagged monomer SOAR or dimer–concatemer SOAR, we used the Leica DMI 6000B inverted automated fluorescence microscope equipped with CFP (438Ex/483Em), YFP (500Ex/542Em) and FRET (438Ex/542Em) filter cubes. At each time point, three sets of images (CFP, YFP and FRET) were collected at room temperature using a × 40 oil objective (numerical aperture 1.35; Leica) and processed using Slidebook 6.0 software (Intelligent Imaging Innovations). Images were captured at 20-s intervals. Exposure times for the CFP, YFP and FRET channels were 1,000, 250 and 1000, ms, respectively. The decreased YFP channel exposure time compensates for the greater fluorescence intensity of YFP compared with CFP. Three-channel-corrected FRET was calculated using the formula:


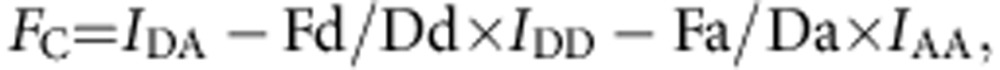


where *I*_DD_, *I*_AA_ and *I*_DA_ represent the background subtracted CFP, YFP and FRET images, respectively, *F*_C_ represents the corrected energy transfer, Fd/Dd represents measured bleed-through of CFP through the FRET filter (0.457) and Fa/Da is the bleed-through of YFP through the FRET filter (0.19). We used the E-FRET method to analyse 3-cube FRET images as describe by Zal and Gascoigne^25^, using the formula:


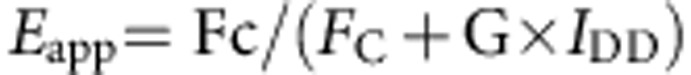


where G is the instrument specific constant^25^,^36^. The EYFP-ECFP construct made as described above, was used to determine the G-parameter for E-FRET calculations. The value of G was determined by measuring the CFP fluorescence increase after YFP acceptor photobleaching using HEK-WT cells transiently transfected with the pEYFP-ECFP construct. The value of G was calculated as 1.9±0.1 (*n*=32 cells). In experiments measuring E-FRET between YFP- and CFP-constructs, cells were selected with a narrow range of YFP/CFP ratios to ensure comparability between measurements. To assure we did not saturate Orai1-CFP (or PM-CFP-Orai1CT) with SOAR concatemer–dimers, we restricted the YFP/CFP ratio to ≤1.0. We calibrated the relative YFP/CFP fluorescence ratio using a STIM1 construct with both N-terminal YFP and C-terminal CFP tags. Expressed in HEK cells, the ratio of YFP/CFP fluorescence was measured in cells in which stores were emptied with ionomycin to assure minimal FRET between YFP and CFP. Measured under identical conditions as the E-FRET measurements above, the YFP/CFP ratio was 1.33±0.04. For near-PM E-FRET measurements, fluorescence data were restricted by selecting the near-PM cell region as described by Navarro-Borelly *et al*^36^.

### Enhanced fluorescence image analysis

Enhanced images of the cellular distribution of fluorescently tagged SOAR, or concatemer–dimer SOAR and Orai1 constructs were obtained from stacks of 10–20 three-dimensional *z* axis image planes collected at 1-μm steps. The constrained iterative deconvolution function of the Slidebook 6.0 software was used to analyse image stacks and derive enhanced deconvolved images with minimized fluorescence contamination from out-of-focus planes^34^. SOAR and Orai1 images shown were typical of at least three independent experiments.

### Electrophysiological measurements

Patch-clamp recording was performed on the stable HEK-Orai1-CFP cell line transiently transfected 24 h prior with monomer or concatemer–dimer SOAR constructs described, based on earlier methodology^37^. The pipette solution contained (in mM):135 Cs-Aspartate, 10 HEPES, 4 MgCl_2_, 10 EGTA and 3.6 CaCl_2_ (pH 7.2 with CsOH). Quantities of EGTA and CaCl_2_ were calculated using WEBMAXCLITE (http://web.stanford.edu /∼cpatton/webmaxc2) to maintain cytosolic Ca^2+^ at ∼90 nM throughout experiments. The bath solution contained (in mM): 130 NaCl, 4.5 KCl, 5 HEPES, 10 Dextrose, 10 TEA-Cl and 20 CaCl_2_ (pH 7.4 with NaOH). Currents were recorded in the standard whole-cell configuration using an EPC-10 amplifier (HEKA). Glass electrodes with typical resistance of 2–4 MΩ were pulled using a P-97 pipette puller (Sutter Instrument). A 50-ms step to −100 mV from a holding potential of 0 mV, followed by a 50-ms ramp from −100 to 100 mV, was delivered every 2 s. The current measured at −100 mV was used in *I*–*V* curves. Currents were filtered at 3.0 kHz and sampled at 20 kHz. A +10-mV junction potential compensation was applied to correct the liquid junction potential between the pipette and bath solutions. All data were acquired using PatchMaster and later analysed using FitMaster and Prism.

### Western analyses

Cells were lysed in pre-chilled lysis buffer containing 150 mM NaCl, 10 mM Tris-HCl (pH 7.4), 1% NP-40 and one tablet of complete protease inhibitors (Santa Cruz, sc-29131) per 25 ml. Lysis was on ice for 30 min, followed by centrifugation at 14,000*g*, 4 °C for 10 min. Supernatant protein was quantified using Bio-Rad DC protein assay kits. Protein extracts (27 μg per lane) were resolved on 4–12% NuPAGE Bis-Tris precast gels (Life Technologies) and transferred to Bio-Rad Immun-Blot PVDF membranes (162-0177, Bio-Rad). After transfer, polyvinylidene difluoride membranes were blocked in PBS–Tween 20 (PBST, containing 1 × PBS, 0.1% Tween 20) containing 5% non-fat dry milk (M0841, LabScientific) for 1 h at room temperature, and then incubated with mouse anti-GFP primary antibody at a final concentration of 1 μg ml^−1^ (Santa Cruz, catalogue number SC9996,) overnight at 4 °C. Membranes were washed three times (7 min) in PBST at room temperature and incubated with secondary antibody (anti-mouse immunoglobulin G from GE healthcare, NA931V) diluted 1:4,000, for 30 min at room temperature. Subsequently, membranes were washed three times (5 min) in PBST. Peroxidase activity was measured using SuperSignal West Pico Chemiluminescent Substrate (Thermo Scientific) following the manufacturer's protocols; the resulting chemifluorescence was imaged using the FluorChem M imager from ProteinSimple.

### Size-exclusion chromatography and crosslinking

The SOAR and SOAR-F394H proteins were prepared as previously described^10^. Purified SOAR and SOAR-F394H proteins were concentrated to ∼0.5 mg ml^−1^ in buffer containing 200 mM NaCl with 20 mM Mes-NaOH, pH 6.5. Samples were loaded onto a Superdex-200 16/60 (GE Healthcare) column previously equilibrated with the same buffer using an AKTA FPLC system (GE Healthcare). Protein was eluted at a flow rate of 2.5 ml min^−1^ and monitored via absorbance at 280 nm. Fractions were collected via an automated fraction collector. Protein standards shown (Bio-Rad) were loaded onto the column for molecular weight calibration using the same method. Purified proteins were dialysed against PBS at room temperature for 2 h. Proteins were concentrated to 0.5 mg ml^−1^ and treated with disuccinimidyl suberate crosslinker (Pierce; 0, 0.1, and 1 mm) for 30 min at room temperature. Reactions were terminated by adding 1 M Tris-HCl (pH 7.5) at a final concentration of 50 mM, and analysed by SDS–polyacrylamide (12%) gel electrophoresis. Protein was stained with Coomassie blue.

### Fluorescence protease protection assay

To confirm the orientation and membrane topology of PM-CFP-Orai1CT, we undertook a fluorescence protease protection assay^29^. Briefly, HEK cells stably expressing PM-CFP-Orai1CT, were treated with 50 μM digitonin in KHM solution (110 mM potassium acetate, 20 mM HEPES and 2 mM MgCl_2_) for 7 min. Control cells were incubated with KHM solution alone for 7 min. Fluorescence images were collected by a Leica DMI 6000B fluorescence microscope equipped with Hamamatsu ORCA-Flash 4 camera controlled using Slidebook 6.0 software (Intelligent Imaging Innovations; Denver, CO) immediately following after preincubation. To minimize photobleaching, the imaging interval was set to 10 s. A amount of 4 mM trypsin was applied after 60 s. All experiments were performed at room temperature and representative traces of at least three independent repeats are shown as mean±s.e.m.

## Additional information

**How to cite this article**: Zhou, Y. *et al.* STIM1 dimers undergo unimolecular coupling to activate Orai1 channels. *Nat. Commun.* 6:8395 doi: 10.1038/ncomms9395 (2015).

## Supplementary Material

Supplementary Information Supplementary Figures 1-6 and Supplementary References

## Figures and Tables

**Figure 1:**
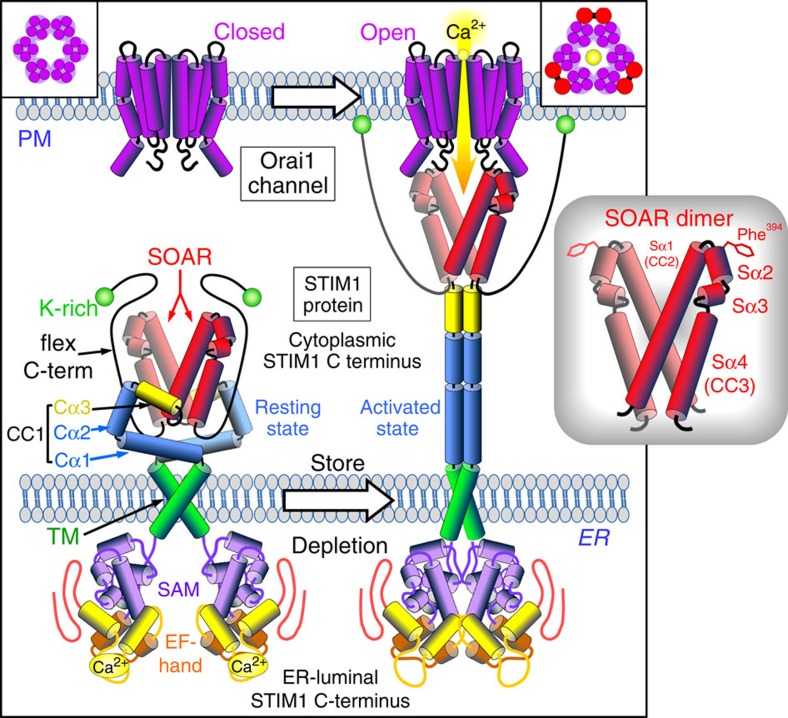
Molecular model of the activation and coupling of STIM1 to activate the Orai1 channel. The dimeric, single transmembrane-spanning STIM1 ER protein senses ER luminal Ca^2+^ change through its N-terminal complex of EF hands and sterile-α motif (SAM). The STIM1 cytoplasmic C terminus is a largely helical complex including the CC1 region, the STIM-Orai-activating region (SOAR), and a flexible C terminus ending with a K-rich region. In the STIM1-resting state, the EF hand/SAM domains are separated, and the SOAR region is occluded within a folded complex of SOAR, CC1 and the flexible C terminus. On luminal Ca^2+^ store depletion, Ca^2+^ dissociation from the EF hand/SAM domains allows the N termini to associate forcing a conformational change in the STIM1 C terminus mediated through rearrangement of the TM domains. The STIM1 C terminus undergoes unfolding and elongation through dissociation between the CC1 and SOAR regions. The extended, unfolded C terminus allows the K-rich C termini of STIM1 to bind to the PM, and also exposes SOAR in this extended configuration allowing it to associate with PM Orai1 channels within closely membrane-associated ER–PM junctions. Orai1 is a four transmembrane-spanning PM protein, forming hexameric Ca^2+^ channels (box, left). Orai1 channels can be tethered within ER–PM junctions by the extended, exposed SOAR unit of activated STIM1. SOAR binding to Orai1 induces gating of the channel to allow Ca^2+^ entry. On the basis of recent information, the model depicts interaction between the SOAR dimer and two adjacent Orai1 subunits of the channel hexamer (box, top right). A more detailed diagram of the molecular organization of the SOAR dimer (inset, right) is from the crystal structure^10^. Each monomer has four α-helices, interacting together through hydrophobic and hydrogen bonding at three interfaces. The two larger helices, Sα1 and Sα4 (also known as CC2 and CC3, respectively), flank two smaller helices (Sα2 and Sα3). The apical Sα2 helix includes an exposed residue, phenylalanine-394, shown to be critical to the binding and gating of Orai1 channels by STIM1 (ref. 20).
