# Supplementary material for: STIM1 dimers undergo unimolecular coupling to activate Orai1 channels
Source: Nat Commun. 2015 Sep 24;6:8395. doi: 10.1038/ncomms9395 (PMC4598629; doi:10.1038/ncomms9395)
Supplement: Supplementary Information — Supplementary Figures 1-6 and Supplementary References [file ncomms9395-s1.pdf]

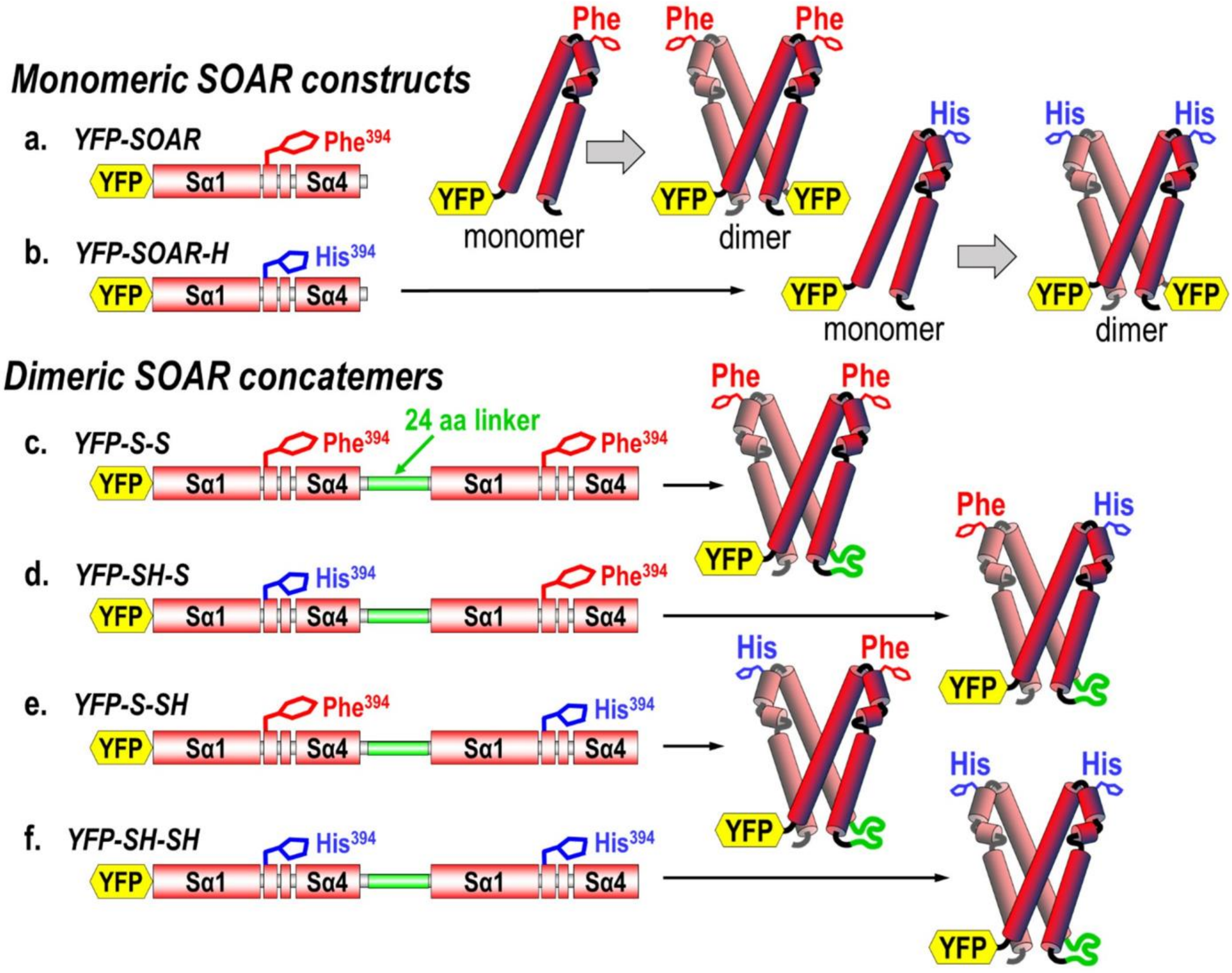

Supplementary Figure 1 | SOAR constructs used in studies. "Monomeric" SOAR constructs are YFP-tagged at the N-terminus, and include, (**a**) the wildtype SOAR protein (YFP-SOAR) or, (**b**) the single point-mutated (F394H) version of this construct (YFP-SOAR-H). Both SOAR proteins exist as dimers when expressed. "Dimeric" SOAR concatemer constructs included two tandem repeats of SOAR joined by a 24-amino acid linker (GGSGGSGGGILQSR-GGSGGSGGSG). These concatemer-dimer dimeric constructs comprised either, (**c**) two concatenated wildtype SOAR units (YFP-S-S), (**d**) a concatemer of N-terminal mutant F394H SOAR and C-terminal wildtype SOAR (YFP-SH-S), (**e**) a concatemer of N-terminal wildtype SOAR and C-terminal F394H SOAR (YFP-S-SH), or (**f**) a concatemer of two F394H mutant SOAR units (YFP-S-SH).

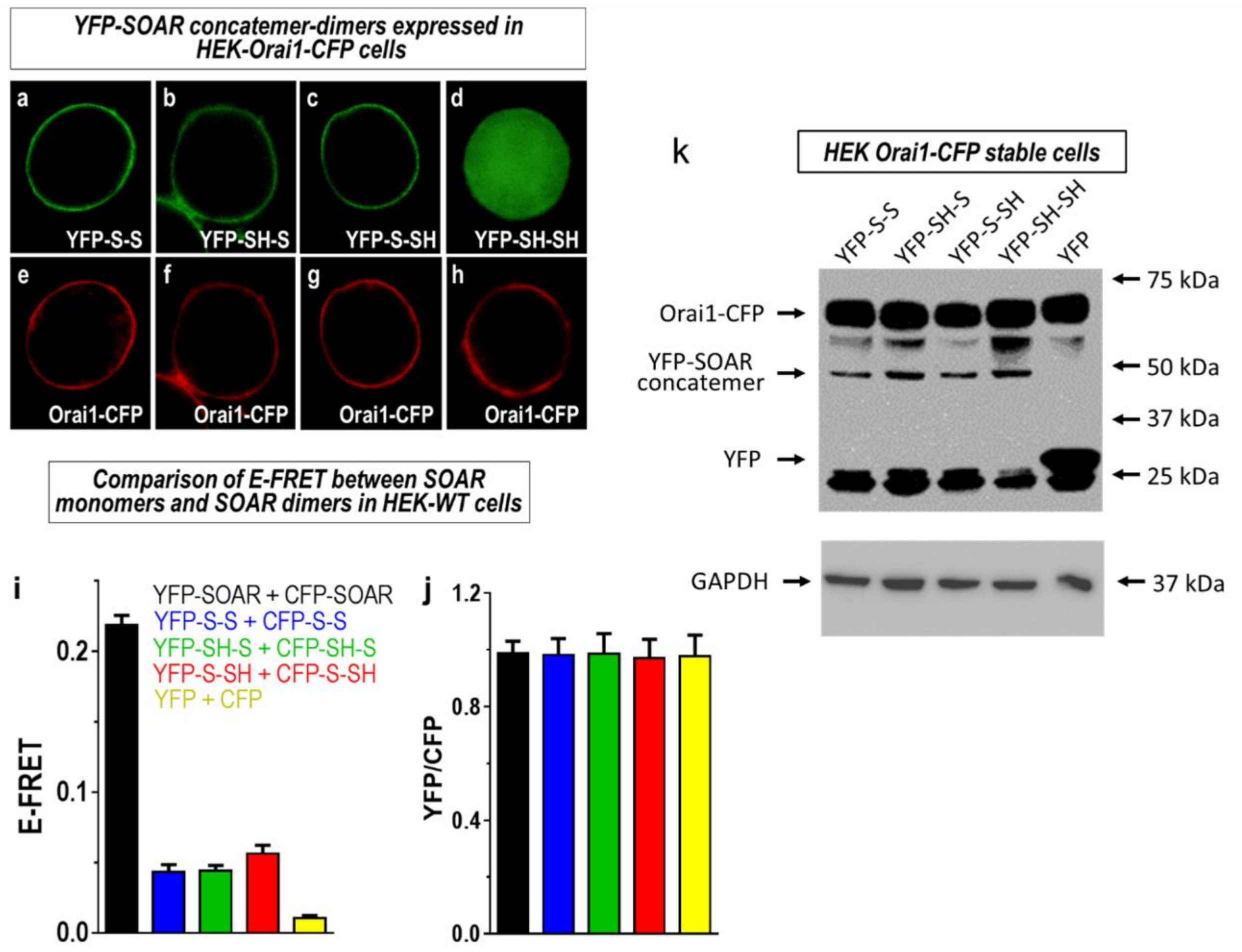

Supplementary Figure 2 | Expression of dimeric SOAR concatemers and FRET comparison for SOAR monomer and dimer interactions. (**a-h**) Comparison of distribution of YFP-tagged SOAR concatemers (**a-d**) and Orai-CFP (**e-h**) in the same cells shown in Fig. 3g-j. The four SOAR YFP-tagged concatemer-dimers were each transiently expressed in stable HEK Orai1-CFP cells: (**a,e**) YFP-S-S and Orai1-CFP distribution in the same cell; (**b,f**) YFP-SH-S and Orai1-CFP distribution in the same cell; (**c,g**) YFP-S-SH and Orai1-CFP distribution in the same cell; (**d,h**) YFP-SH-SH and Orai1-CFP distribution in the same cell. (**i,j**) Comparison of whole cell E-FRET measurements between monomeric SOAR or between SOAR concatemer-dimers. (**i**) YFP-SOAR and CFP-SOAR co-expressed in HEK-WT cells (red; n=81); YFP-S-S and CFP-S-S co-expressed in HEK-WT cells (green; n=16); unconjugated YFP and CFP co-expressed in HEK-WT cells (blue; n=68). (**j**) YFP/CFP ratios of the whole cell-expressed CFP and YFP constructs used for E-FRET in (**i**). (**k**) Western analysis of expression of YFP-S-S, YFP-SH-S, YFP-S-SH and YFP-SH-SH in HEK Orai1-CFP stable cells detected with GFP antibody, and compared with GAPDH expression. Results are means ± SEM.

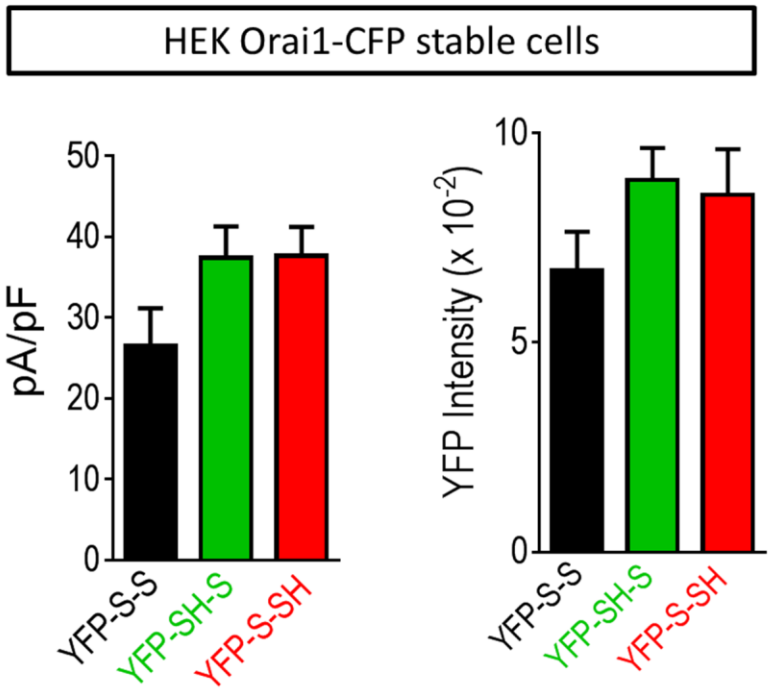

Supplementary Figure 3 | Summary data for current density measurements shown in Fig. 4h. Results are means ± SEM: n=22 (YFP-S-S); n=21 (YFP-SH-S); n=19 (YFP-S-SH). Left shows the actual pA/pF summary values. Right shows the average YFP fluorescence intensity for the same experiments. Note, that the correlation between the two sets of values is high, therefore, the pA/pF values corrected for fluorescence are very similar.

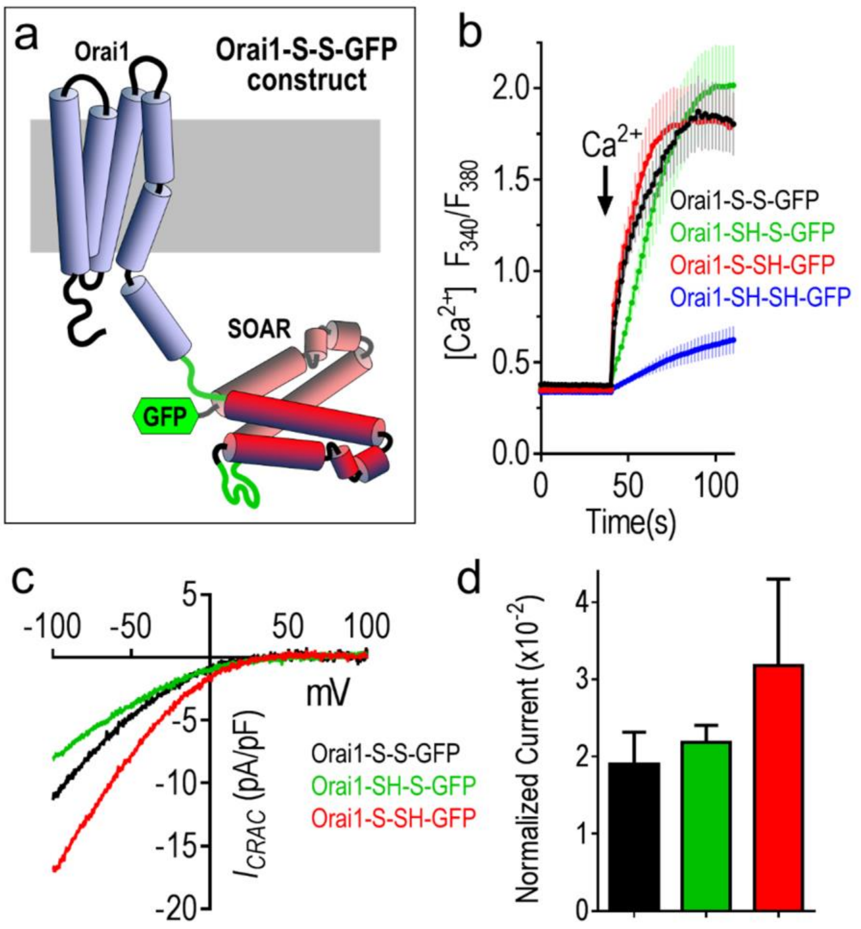

Supplementary Figure 4 | Ca^{2+} entry and CRAC current mediated by the Orai1-SOAR-SOAR concatemer (Orai1-S-S). (a) The concatemeric Orai1-S-S comprising the entire Orai1 channel concatenated with two SOAR dimers tagged at the C-terminus with GFP (Orai1-S-S-GFP). The F394H mutation was introduced into one or both SOAR units to give Orai1-SH-S-GFP, Orai1-S-SH-GFP, and Orai1-SH-SH-GFP, according to the notation in Supplementary Fig. 1c-1f. (b) Fura-2 measurement of constitutive Ca^{2+} entry in wildtype HEK cells expressing Orai1-S-S-GFP (black), Orai1-SH-S-GFP (green), Orai1-S-SH-GFP (red), and Orai1-SH-SH-GFP (blue). Note, all three constructs containing at least one wildtype SOAR unit, gave rise to similar Ca^{2+} entry. Results are means ± s.e.m. of 15-30 cells per field. (c) I/V relationship of whole-cell I_{CRAC} measurements in wildtype HEK cells expressing Orai1-S-S-GFP (black), YFP-SH-S (green), or YFP-S-SH (red). (d) Summary of peak current amplitudes for Orai1-S-S-GFP (black, n=4), YFP-SH-S (green, n=6), or YFP-S-SH (red, n=3). Current values are means ± SEM values normalized to GFP fluorescence intensity to avoid any variation caused by different expression levels.

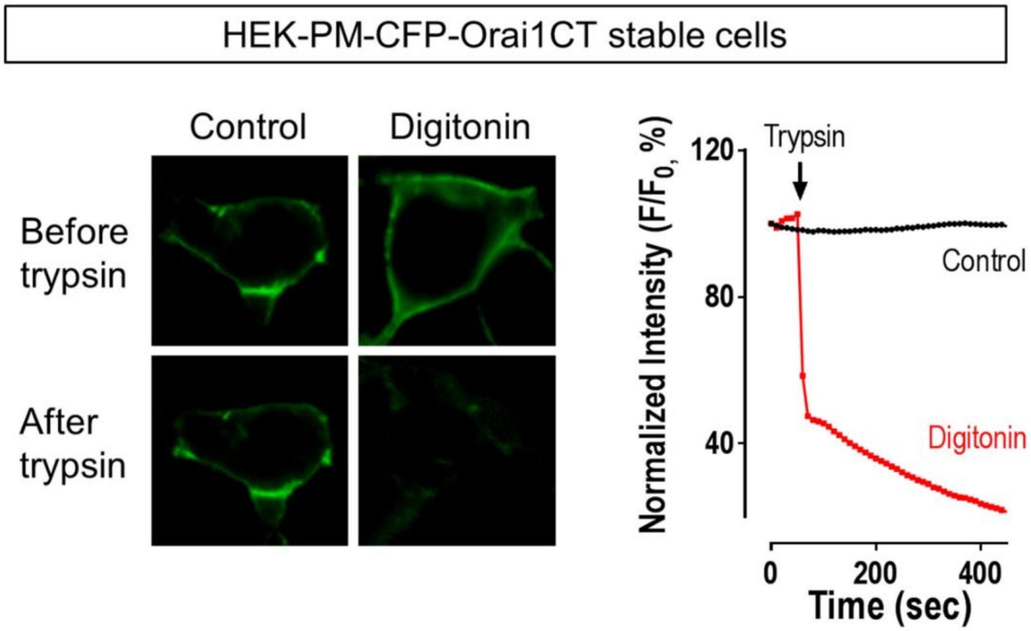

Supplementary Figure 5 | Determination of PM-septicity and orientation of the PM-CFP-Orai1CT construct, using a fluorescence protease protection (FPP) assay. HEK cells stably expressing PM-CFP-Orai1CT, were treated with 50 µM digitonin in KHM solution for 7 min. Control cells were incubated with KHM solution alone for 7 min. Fluorescence images were collected immediately following preincubation. 4 mM trypsin was applied after 60 seconds. Experiments were performed at room temperature and representative traces of at least three independent repeats are shown as means ± SEM.

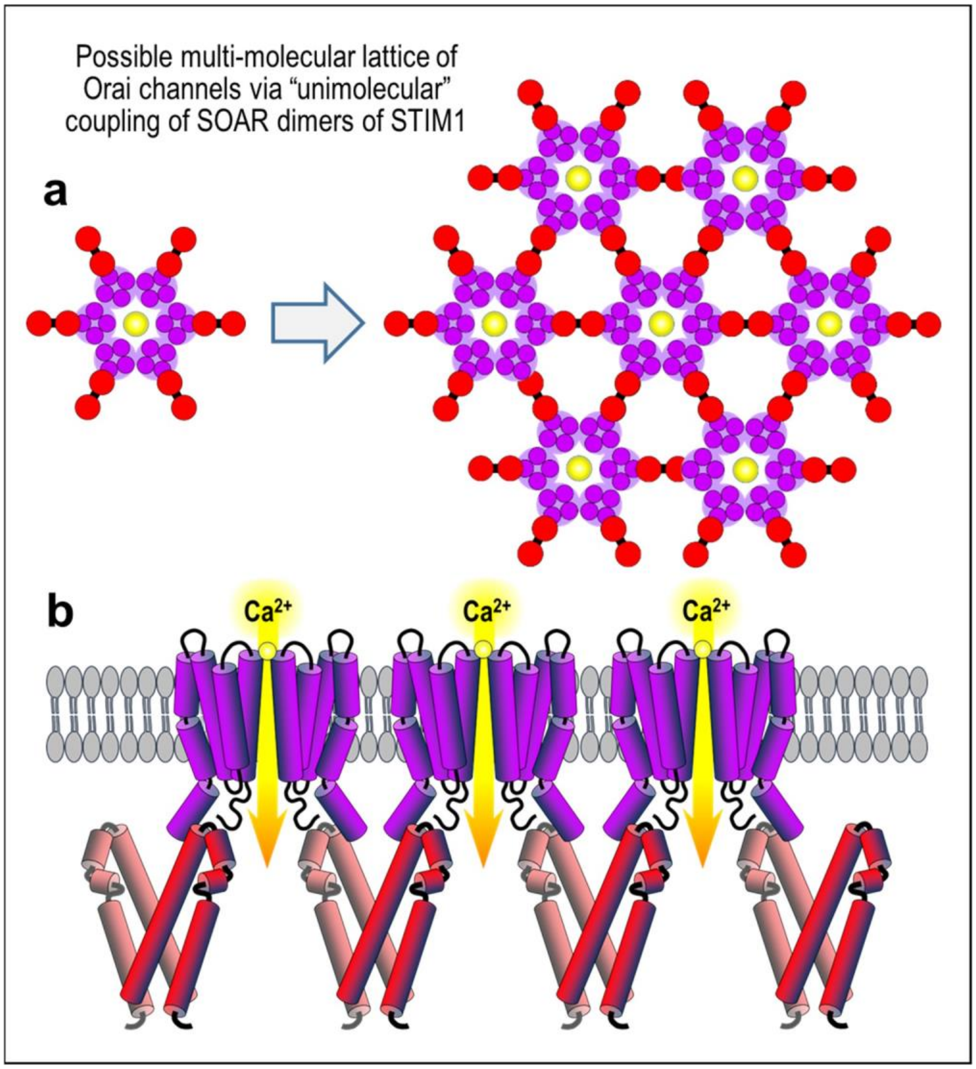

Supplementary Figure 6 | Model depicting hypothetical clustering of Orai1 channels by SOAR dimers. Evidence from previous work revealed the SOAR-like CAD molecule from STIM1 could cross-link Orai1 channels into clusters[1]. Based on the current evidence that dimeric SOAR molecules undergo unimolecular coupling to bind to and activate Orai1 channels, and based on evidence that dimeric SOAR molecules can simultaneously bind two Orai1 C- terminal STIM1-binding domains, we speculate that the exposed dimeric SOAR unit on activated and unfolded STIM1 molecules could mediate cross-linking between channels. Without clustering, maximal channel activation would occur with a STIM1:Orai1 ratio of 2:1, that is, 12 STIM1 proteins (6 STIM1 dimers) bound to 6 Orai1 proteins (a single Orai1 channel). If cross-linking occurred on a large scale (for example, with hundreds of Orai channels in a single cluster), the STIM1:Orai1 ratio in a single cluster would approach 1. In a smaller cluster, for example that shown in (**a**) with 7 Orai channels, the STIM1:Orai1 ratio would be 1.43, assuming

all Orai1 binding sites were occupied by STIM1. A further interesting corollary to the unimolecular coupling model is that the STIM1 binding site on the Orai1 channel is likely small. The complete failure of the STIM1 F394H mutant to couple to Orai1 indeed suggests a limited interaction site. The F394 residue is midway between the larger Sα1 and Sα4 helices in a relatively unstructured region. Unlike the R429C mutation, the F394H mutation does not cause substantial unfolding or activation of STIM1, hence it may be more likely that F394 represents an important local molecular component in the Orai1 coupling event. We speculate in (**b**) that the site of interaction with SOAR may involve both the N- and C-termini of Orai1 as has been suggested[1-3], but we also suggest that the interacting components may be very limited.

References to Supplementary Figures.

1. Park, C. Y. *et al.* STIM1 clusters and activates CRAC channels via direct binding of a cytosolic domain to Orai1. *Cell* **136**, 876-890 (2009).
2. Muik, M. *et al.* Dynamic coupling of the putative coiled-coil domain of ORAI1 with STIM1 mediates ORAI1 channel activation. *J. Biol. Chem.* **283**, 8014-8022 (2008).
3. McNally, B. A., Somasundaram, A., Jairaman, A., Yamashita, M. & Prakriya, M. The C- and N-terminal STIM1 binding sites on Orai1 are required for both trapping and gating CRAC channels. *J Physiol* (2013).
